# Early net ultrafiltration thresholds and mortality in critically ill patients with septic acute kidney injury receiving continuous renal replacement therapy

**DOI:** 10.1080/0886022X.2025.2511277

**Published:** 2025-05-29

**Authors:** Chen Long Zhao, Meng Ya Zhao, Hao Wang, De Yuan Zhi, Xiao Jun Ji, Mei Li Duan, Jin Lin

**Affiliations:** a Department of Critical Care Medicine, Beijing Friendship Hospital, Capital Medical University, China; b Department of Clinical Epidemiology and Evidence-Based Medicine, National Clinical Research Center for Digestive Disease, Beijing Friendship Hospital, Capital Medical University

**Keywords:** Net ultrafiltration, continuous renal replacement therapy, sepsis, acute kidney injury

## Abstract

**Background:**

Net ultrafiltration (NUF) rates correlate with outcomes in critically ill patients on continuous renal replacement therapy (CRRT), but optimal strategies for septic acute kidney injury (AKI) are unclear. This study evaluated early NUF rates and survival in septic AKI.

**Methods:**

A retrospective cohort of 219 adults with septic AKI requiring CRRT at a tertiary ICU was analyzed. Early NUF (weight-adjusted fluid removal/hour during the first 48 h of CRRT) was stratified into low- (<1.22 mL/kg/h), moderate- (1.22–1.79 mL/kg/h), and high-intensity (>1.79 mL/kg/h) groups. The primary outcome was 28-day mortality. Associations were assessed using multivariable Cox regression and restricted cubic spline models, adjusted for demographics, severity scores, fluid balance, and biomarkers.

**Results:**

The high-intensity group had the highest 28-day mortality (68.5% vs. 43.8% moderate vs. 45.2% low). High-intensity NUF was independently associated with increased mortality vs. moderate (adjusted HR = 1.88, 95% CI:1.19–2.97, *p* = 0.007) and low-intensity groups (adjusted HR = 2.01, 95% CI:1.25–3.22, *p* = 0.004). Nonlinear analysis demonstrated a nonlinear relationship, with risks escalating steeply at rates above 1.79 mL/kg/h.

**Conclusion:**

High-intensity NUF during early CRRT was associated with higher mortality in patients with septic AKI mortality, particularly among those with high severity of illness. Moderate NUF had lowest mortality, suggesting that intermediate NUF rates may best balance the competing risks of worsening hemodynamic instability from excess NUF and persistent volume overload from inadequate NUF. However, future trials are needed to better define the optimal approach to NUF in patients with septic AKI.

## Introduction

Acute kidney injury (AKI) in the context of sepsis presents substantial clinical challenges, with mortality rates exceeding 40% among critically ill patients requiring renal replacement therapy [1–3]. Continuous renal replacement therapy (CRRT) has become the preferred modality for hemodynamically unstable patients [4], yet critical uncertainties remain regarding optimal fluid management strategies, particularly the prescription of net ultrafiltration (NUF) rates.

Recent evidence indicates that cumulative fluid balance before and during CRRT independently predicts mortality in septic AKI [5–7]. Although Ostermann et al. demonstrated improved survival with net fluid removal [8], their analysis focused on cumulative balance rather than NUF prescription—the principal clinician-controlled variable governing fluid extraction during CRRT. Mechanistically, NUF influences both volume status and circulatory stress *via* two opposing pathways: correcting fluid overload (which enhances tissue oxygenation) and inducing hypovolemia (which exacerbates organ hypoperfusion) [9]. This equilibrium is especially unstable in septic AKI, where altered capillary permeability and vasoplegia promote pathological fluid redistribution [10].

Clinical studies yield conflicting recommendations. Single-center analyses associate slower NUF rates (<1.01 mL/kg/h) with increased mortality, while *post hoc* evaluations of the RENAL trial suggest harm from faster rates (>1.75 mL/kg/h) [11,12]. These apparent discrepancies may arise from population heterogeneity: previous cohorts included <30% sepsis patients [12–14], potentially masking etiology-specific responses. Inappropriate NUF prescriptions may lead to adverse events such as hemodynamic instability or persistent tissue edema, further exacerbating organ dysfunction [15–18], especially during the critical initial phase of CRRT. However, evidence on optimal NUF intensity for septic AKI remains scarce. This study aimed to assess the impact of different early net ultrafiltration intensities on outcomes in septic AKI patients requiring CRRT.

## Methods

### Study design and population

This retrospective cohort study enrolled adult patients (≥ 18 years) with septic AKI requiring CRRT at a tertiary medical-surgical ICU in Beijing, China, from January 2016 to October 2021. Exclusion criteria included: (1) preexisting end-stage renal disease on maintenance dialysis; (2) prior renal replacement therapy; (3) CRRT duration <24 h; (4) death within 48 h of CRRT initiation; and (5) missing pre-CRRT fluid balance data.

### Data collection

At CRRT initiation, baseline data were collected on demographics (age, sex, height, weight) and clinical variables (diagnostic classification, admission source, comorbidities, infection site), pre-CRRT time variables (ICU-to-CRRT interval; 24-h urine output; cumulative fluid balance; AKI stage), CRRT indications, hemodynamic status (mean arterial pressure, septic shock), and mechanical ventilation. Disease severity was assessed using Sequential Organ Failure Assessment (SOFA) scores and Acute Physiology and Chronic Health Evaluation II (APACHE II) scores calculated at CRRT commencement. Laboratory variables comprised complete blood count (white blood cells, hemoglobin, platelets), serum electrolytes (sodium, potassium), renal/liver function markers (creatinine, total bilirubin, albumin), inflammatory biomarkers (procalcitonin), acid-base status (pH), P/F ratio (PaO_2_/FiO_2_), lactate levels, and N-terminal pro-B-type natriuretic peptide (NT-proBNP). CRRT metrics included daily therapy duration (hours) and net ultrafiltration volume (mL).

### Definition

We defined AKI based on the Kidney Disease: Improving Global Outcomes (KDIGO) clinical practice guidelines. AKI was diagnosed by meeting at least one of the following three criteria: 1. An increase in the serum creatinine (sCr) level ≥ 0.3 mg/dl (26.5 µmol/L) within 48 h; 2. An increase in the sCr level to ≥ 1.5 times the baseline value (known or presumed to have occurred within the preceding 7 days); 3. Urine output < 0.5 mL/kg/h for 6 consecutive hours [19].

The diagnosis of sepsis and septic shock followed the 2016 International Consensus Definitions (Sepsis-3) [20]. Sepsis was defined as suspected infection concurrent with a SOFA score increase ≥2 points from baseline. Septic shock was characterized by sepsis requiring vasopressors to maintain mean arterial pressure ≥65 mmHg, despite adequate fluid resuscitation, and serum lactate >2 mmol/L. Septic AKI was defined as acute renal function deterioration occurring in the context of sepsis or septic shock.

To normalize individual fluid status, cumulative fluid balance (CFB) values were divided by admission body weight (kg) and converted into percentage weight-adjusted CFB.

### Determination of NUF intensity

NUF denotes the volume of fluid extracted from a patient’s bloodstream during renal replacement therapy within the first 48 h after CRRT initiation. In this study, the exposure variable was the NUF rate, defined as the adjusted volume of NUF per hour based on patient weight. The formula for calculating the net ultrafiltration rate was as follows:

$$\begin{array}{l} \text{Net Ultrafiltration Rate}\left(\text{ml}/\text{kg}\cdot h\right)\\ =\frac{\text{Cumulative}\;\text{Net}\;\text{Ultrafiltration}\;\text{Volume}\left(\text{ml}\right)}{\text{Weight}\;\text{at}\;\text{admission}\;\text{to}\;\text{ICU}\left(\text{kg}\right)*\text{CRRT}\;\text{Duration}\left(h\right)} \end{array}$$

This net ultrafiltration rate may also be considered machine net ultrafiltration rate (i.e. fluid balance with respect to the CRRT device) as it does not account for any fluid input into or fluid output from the patient outside the CRRT circuit; this is not to be confused with patient fluid balance, which has been also termed patient net ultrafiltration.

### Study outcome

The primary outcome was all-cause mortality at 28 days after CRRT initiation. The secondary outcomes included 60-day all-cause mortality, cumulative fluid balance at 72 h, change in SOFA score at 72 h after CRRT initiation (ΔSOFA), the length of ICU stay and dependence on renal replacement therapy at ICU discharge.

### Statistical analysis

Based on preliminary exploratory analyses, this study stratified participants into tertiles by NUF rates during the first 48 h of CRRT: low-intensity (<1.22 mL/kg/h), moderate-intensity (1.22–1.79 mL/kg/h), and high-intensity (>1.79 mL/kg/h) groups. Continuous variables were presented as mean ± standard deviation or median (interquartile range); comparisons between groups were performed using one-way ANOVA (for normally distributed data) or Kruskal–Wallis tests (for nonparametric data). Categorical variables were summarized as frequencies (percentages), with group differences examined by chi-square tests.

Kaplan–Meier survival curves were generated to evaluate 28-day mortality, and log-rank tests were used to compare survival distributions. Cox proportional hazards regression models were employed to investigate associations between NUF intensity and mortality. Covariates were selected based on statistical significance (*p* < 0.1 in univariate analysis) and clinical relevance. Prior to multivariable analyses, collinearity diagnostics were performed, and the Box–Tidwell method was applied to verify linearity assumptions for continuous variables. We constructed three sequential models: Model 1 adjusted for demographics (age and sex); Model 2 added disease severity markers (septic shock, mechanical ventilation, SOFA and APACHE II scores); Model 3 further included weight-adjusted cumulative fluid balance, baseline creatinine, and lactate levels. A Bonferroni-corrected significance threshold of *p* < 0.0167 (0.05/3) was applied to account for multiple comparisons. Restricted cubic spline (RCS) models were constructed to visualize dose-response relationships between NUF rates (analyzed as continuous variables) and mortality outcomes.

To ensure robustness of findings, sensitivity analyses were performed in three sequential phases: First, multivariable logistic regression models generated adjusted odds ratios (aORs) for mortality; Second, refined models retained predictors with the smallest p-values from the univariate screening; Third, primary analyses were replicated using 72-h NUF volumes (instead of 48-h data) for group reclassification. Finally, stratified Cox regression analyses evaluated subgroup heterogeneity across four high-risk categories: (1) septic shock, (2) pre-CRRT fluid overload ≥5%, (3) SOFA score >8, and (4) lactate >2 mmol/L. Bonferroni correction adjusted significance thresholds for multiple comparisons.

Statistical analyses utilized SPSS 22.0, R 4.0.1, and STATA 14.1. All tests were two-tailed, with *p* values <0.05 considered statistically significant.

## Results

### Baseline characteristics

**Figure 1.**
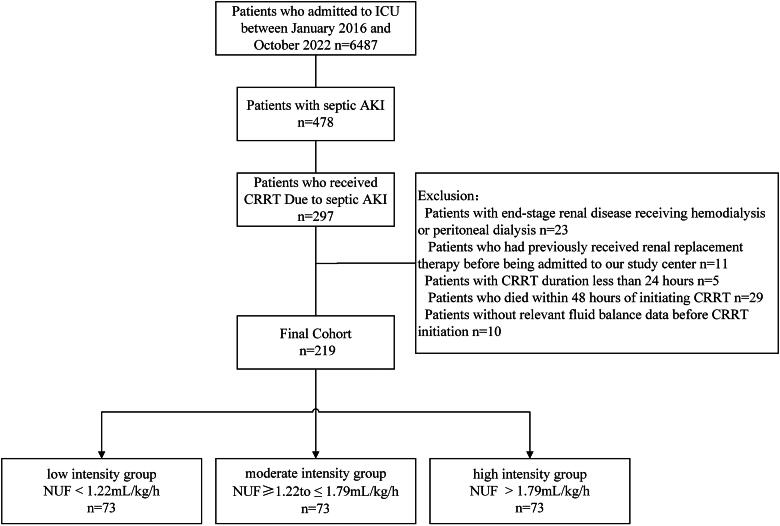
Flowchart of patient enrollment. AKI, Acute Kidney Injury; ICU, Intensive Care Unit; CRRT, Continuous Renal Replacement Therapy; NUF, Net Ultrafiltration

During the 5-year observational period, 6,487 critically ill adult patients were admitted to the ICU, of whom 297 developed septic AKI requiring CRRT. After applying predefined exclusion criteria (Figure 1), the final cohort consisted of 219 patients.

The cohort’s baseline demographic, clinical, and laboratory characteristics, stratified by tertiles of NUF intensity (low: <1.22 mL/kg/h; moderate: 1.22–1.79 mL/kg/h; high: >1.79 mL/kg/h), are detailed in Table 1. The majority of participants were male (65.8%, 144/219) with a mean age of 66.3 ± 18.6 years. Patients in the high-intensity NUF group exhibited distinct clinical features, including a higher proportion of female patients, lower body weight, reduced body mass index (BMI), and elevated NT-proBNP levels. In contrast, the low-intensity group demonstrated higher hemoglobin concentrations and better P/F ratio. No significant differences were observed among the three groups in other variables.

**Table 1.** Comparison of baseline characteristics by net ultrafiltration (NUF) rate groups.

Variables	All (*n* = 219)	Low-intensity (*n* = 73)	Moderate-intensity (*n* = 73)	High-intensity (*n* = 73)	*p* value
Age (years)	66.3 ± 18.6	66.5 ± 17.9	66.6 ± 19.0	65.8 ± 19.3	0.950
Male n (%)	144 (65.8)	53 (72.6)	57 (78.1)	34 (46.6)	<0.001
Weight (kg)	68.2 ± 16.7	75.8 ± 20.5	69.2 ± 12.4	59.5 ± 11.4	<0.001
BMI (kg/m^2^)	24.0 ± 4.7	26.1 ± 5.5	24.1 ± 3.9	21.8 ± 3.5	<0.001
Baseline Cr (μmol/L)	81.6 (64.0, 109.4)	83.2 (60.7, 80.5)	84.4 (71.2, 113.2)	77.4 (62.1, 114.0)	0.240
Comorbidity, n (%)					
Hypertension	86 (39.3)	29 (39.7)	30 (40.1)	27 (37.0)	0.875
Cardiac disease	67 (30.6)	22 (30.1)	26 (35.6)	19 (26.0)	0.451
Diabetes	77 (35.2)	30 (41.1)	26 (35.6)	21 (28.8)	0.295
Chronic kidney disease	55 (25.1)	15 (20.5)	21 (28.8)	19 (26.0)	0.507
Admission source, n (%)					0.054
Emergency	72 (32.9)	17 (23.3)	24 (32.9)	31 (42.5)	
Medical ward	80 (36.5)	32 (43.8)	20 (27.4)	28 (38.4)	
Surgical ward	60 (27.4)	22 (30.1)	26 (35.6)	12 (16.4)	
Transfer from another hospital	7 (3.2)	2 (2.7)	3 (4.1)	2 (2.7)	
Site of infection, n (%)					0.487
Respiratory	156 (71.2)	57 (78.1)	46 (63.0)	53 (72.6)	
Digestive	36 (16.4)	9 (12.3)	16 (21.9)	11 (15.1)	
Urinary	19 (8.7)	6 (8.2)	6 (8.2)	7 (9.6)	
Blood	4 (1.8)	1 (1.4)	2 (2.7)	1 (1.4)	
Other	4 (1.8)	0 (0)	3 (4.1)	1 (1.4)	
Indications for CRRT, n(%)					0.055
Fluid overload	50 (22.8)	16 (21.9)	13 (17.8)	21 (28.8)	
acid-base disorder	11 (5.0)	7 (9.6)	1 (1.4)	3 (4.1)	
electrolyte disorder	15 (6.8)	5 (6.8)	6 (8.2)	4 (5.5)	
Severe non-renal organ dysfunction	20 (9.1)	7 (9.6)	5 (6.8)	8 (11.0)	
Oliguria	84 (38.4)	31 (42.5)	29 (39.7)	24 (32.9)	
Other	39 (17.8)	7 (9.6)	19 (26)	13 (17.8)	
AKI stage at CRRT initiation, n (%)					0.272
1	46 (21.0)	16 (21.9)	14 (19.2)	16 (21.9)	
2	49 (22.4)	22 (30.1)	12 (16.4)	15 (20.5)	
3	124 (56.6)	35 (47.0)	47 (64.4)	42 (57.5)	
SOFA score	10.7 ± 3.9	10.9 ± 3.9	9.8 ± 3.8	11.3 ± 4.0	0.079
APACHE II score	24.4 ± 6.8	24.1 ± 7.1	24 ± 5.7	25.2 ± 7.6	0.641
Mechanical ventilation, n (%)	153 (69.9)	51 (69.9)	47 (64.4)	55 (75.3)	0.353
Septic shock, n (%)	141 (64.4)	49 (67.1)	42 (57.5)	50 (68.5)	0.321
Time from ICU admission to CRRT initiation (d)	1 (0, 4)	1 (0,2)	1 (0,4)	1 (0,8)	0.617
24-hour urine before CRRT initiation (mL)	442 (250, 720)	440 (237, 817)	425 (240, 717)	465 (370, 697)	0.622
CFB prior to CRRT (mL)	3098 (2040, 4734)	3195 (2250, 4757)	2810 (1948, 4951)	3235 (1983, 4728)	0.758
Weight-adjusted CFB (%) prior to CRRT	4.46 (2.99, 7.87)	4.07 (2.81, 6.81)	4.41 (2.93, 7.67)	5.65 (3.17, 9.10)	0.079
MAP at CRRT initiation	82.6 ± 13.1	83.2 ± 16.0	81.2 ± 12.2	83.4 ± 10.5	0.347
Laboratory before CRRT					
WBC (×10^9^/L)	11.7 (6.7, 16.7)	12.7 (8.8, 17.7)	10.9 (6.6, 14.6)	10.9 (6.2, 19.3)	0.224
Hemoglobin (g/L)	84.0 (72.0, 105.0)	98.0 (81.5, 126.0)	79.0 (68.0, 104.0)	80.0 (66.5, 92.5)	<0.001
Platelets (×10^9^/L)	98.0 (56.0, 164.0)	92.0 (60.0, 151.5)	116.0 (56.0, 183.0)	90.0 (50.5, 160.5)	0.342
Serum sodium (mmol/L)	142.8 ± 8.4	142.8 ± 8.9	142.5 ± 8.6	143.2 ± 7.9	0.967
Serum potassium (mmol/L)	4.66 ± 0.71	4.68 ± 0.80	4.65 ± 0.67	4.64 ± 0.67	0.900
Serum potassium ≥5.5mmol/L	31 (14.2)	9 (12.3)	11 (15.1)	11 (15.1)	0.860
Creatinine (μmol/L)	263.5 (165.3, 431.2)	259.0 (165.6, 409.2)	286.5 (196.0, 464.1)	234.6 (139.3, 437.2)	0.170
Creatinine ≥300 μmol/L	90 (41.1)	32 (43.8)	34 (46.6)	24 (32.9)	0.205
Total bilirubin (μmol/L)	17.8 (10.2, 41.0)	16.0 (10.3, 43.6)	18.2 (9.6, 34.4)	10.0 (10.2, 53.4)	0.781
Albumin (g/L)	26.6 ± 8.7	26.1 ± 4.9	27.9 ± 13.7	26.0 ± 3.8	0.700
Procalcitonin (ng/mL)	4.6 (1.3, 12.4)	5.7 (1.2, 19.5)	3.4 (1.2, 11.9)	5.3 (1.3, 12.3)	0.790
pH value	7.36 ± 0.08	7.36 ± 0.08	7.36 ± 0.09	7.35 ± 0.09	0.897
pH ≤ 7.2	11 (5.0)	1 (1.4)	6 (8.2)	4 (5.5)	0.162
P/F ratio (mmHg)	188 (127, 275)	214 (144, 297)	187 (122, 268)	160 (102, 270)	0.048
Lactate (mmol/L)	1.9 (1.2, 3.4)	2.3 (1.5, 3.4)	1.7 (1.0, 3.1)	2.1 (1.2, 3.5)	0.122
NT-proBNP (pg/mL)	8229 (1859, 28399)	2056 (605, 14663)	10100 (3785, 29360)	17032 (3966, 29358)	<0.001
NUF rate (mL/kg/h)	1.48 (1.05, 2.04)	0.84 (0.58, 1.06)	1.48 (1.35, 1.61)	2.39 (2.03, 2.71)	<0.001

BMI, Body Mass Index; SOFA, Sequential Organ Failure Assessment; APACHE, Acute Physiology and Chronic Health Evaluation; ICU, Intensive Care Unit; CRRT, Continuous Renal Replacement Therapy; CFB, Cumulative Fluid Balance; MAP, Mean Arterial Blood Pressure; WBC, White Blood Cells; P/F ratio, PaO₂/FiO₂ ratio; NUF, Net Ultrafiltration; Continuous variables are expressed as mean ± SD or median [Q1, Q3] and nominal variables as n (%).

### Associations of NUF and mortality

**Figure 2.**
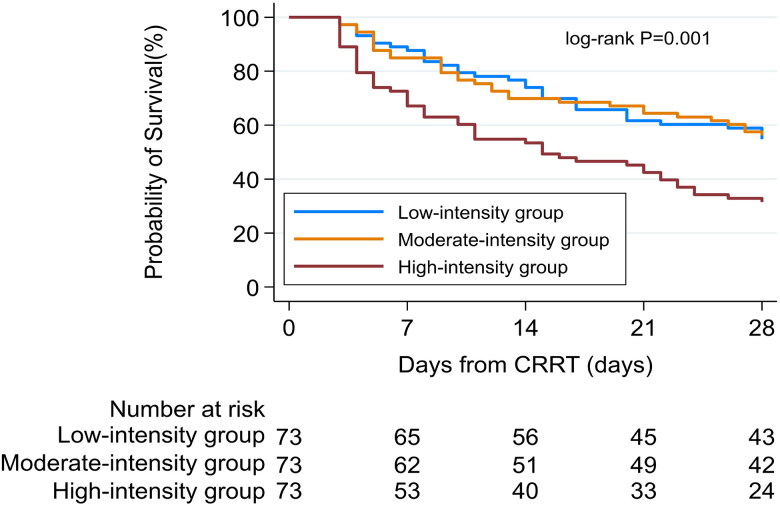
Kaplan-Meier Survival curves for 28-day overall survival after CRRT initiation, stratified by net ultrafiltration (NUF) intensity groups.

During the 28-day period following the initiation of CRRT, a total of 115 deaths (52.5% of the cohort) were recorded. Comparisons of baseline characteristics between survivors and non-survivors are presented in Supplementary Table 1. Survival outcomes differed significantly across NUF tertiles (*p* = 0.004; Table 2). The high-intensity group had the highest 28-day mortality (68.5% [50/73]), which was significantly higher than both the moderate-intensity (43.8% [32/73]) and low-intensity groups (45.2% [33/73]). Kaplan-Meier analysis (Figure 2) demonstrated a survival disadvantage associated with high-intensity NUF (log-rank *p* < 0.001).

**Table 2.** Clinical outcomes stratified by net ultrafiltration (NUF) rate groups.

Outcome	All (*n* = 219)	Low-intensity (*n* = 73)	Moderate-intensity (*n* = 73)	High-intensity (*n* = 73)	*p* value
Primary					
Death at 28 days, n (%)	115 (52.5)	33 (45.2)	32 (43.8)	50 (68.5)	0.004
Secondary					
Death at 60 days, n (%)	127 (58.0)	38 (52.1)	36 (49.3)	53 (72.6)	0.008
CFB at 72 hours after CRRT	538 (−1396, 2361)	2067 (385, 4051)	241 (−1136, 2052)	−1368 (3012, 1181)	<0.001
ΔSOFA score	1.49 ± 4.63	1.15 ± 5.09	1.25 ± 4.47	2.07 ± 4.30	0.588
Length of ICU days					
Survivors	30 (13, 47)	32 (11, 75)	30 (18, 42)	25 (15, 53)	0.903
Non-survivors	10 (5, 17)	12 (7, 17)	10 (5, 20)	8 (4, 17)	0.380
RRT dependence at ICU discharge, n (%)	53 (51.0)	19 (47.5)	22 (53.7)	12 (52.2)	0.850

CFB, Cumulative Fluid Balance; CRRT, Continuous Renal Replacement Therapy; ΔSOFA, change in SOFA at 72 h after CRRT initiation; ICU, Intensive Care Unit; RRT, Renal Replacement Therapy.

In unadjusted models, higher NUF rates correlated with higher mortality risk (high vs. moderate: HR 2.02, 95% CI 1.30–3.15, *p* = 0.002; high vs. low: HR 1.97, 95% CI 1.27–3.06, *p* = 0.003; Table 3). This association persisted after adjusting for age, sex, septic shock, mechanical ventilation, SOFA/APACHE-II scores, weight-adjusted cumulative fluid balance, creatinine, and lactate (adjusted HR [aHR] high vs. moderate: 1.88, 95% CI 1.19–2.97, *p* = 0.007; aHR high vs. low: 2.01, 95% CI 1.25–3.22, *p* = 0.004). The findings remained statistically significant following Bonferroni correction for multiple comparisons (adjusted threshold: *p* < 0.0167).

**Table 3.** Univariate and multivariable Cox regression analyses of 28-day all-cause mortality.

Net ultrafiltration intensity	HR (95% CI)	*p* value	Model 1 aHR (95% CI)	*p* value	Model 2 aHR (95% CI)	*p* value	Model 3 aHR (95% CI)	*p* value
High vs Low	1.97 (1.27–3.06)	0.003	1.85 (1.17–2.94)	0.009	1.99 (1.26–3.14)	0.003	2.01 (1.25–3.22)	0.004
High vs Moderate	2.02 (1.30–3.15)	0.002	1.92 (1.22–3.00)	0.004	1.98 (1.26–3.11)	0.003	1.88 (1.19–2.97)	0.007
Low vs Moderate	1.03 (0.63–1.67)	0.922	1.03 (0.63–1.69)	0.894	0.99 (0.61–1.64)	0.985	0.94 (0.57–1.55)	0.798

HR: Hazard Ratio; aHR: adjusted Hazard Ratio; CI: Confidence Interval.

Model 1 adjusted for age and sex.

Model 2 adjusted for age, sex, presence of septic shock, need for mechanical ventilation, SOFA score, and APACHE II score.

Model 3 adjusted for age, sex, presence of septic shock, need for mechanical ventilation, SOFA score, APACHE II score, weight-adjusted cumulative fluid balance, creatinine, and lactate.

**Figure 3.**
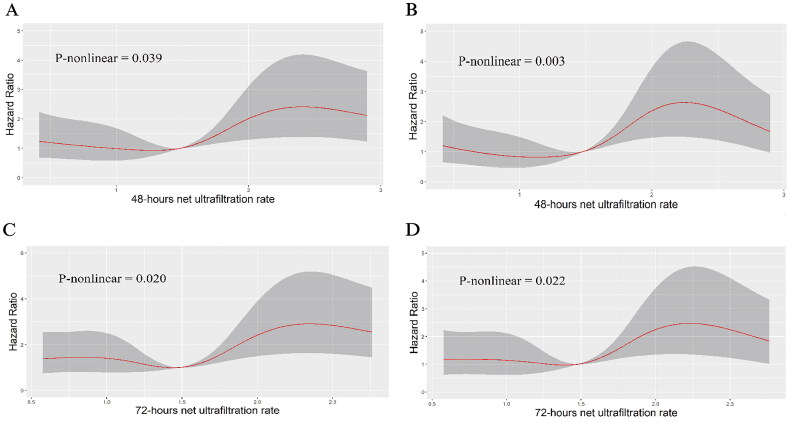
Restricted cubic spline analysis between net ultrafiltration rate (mL/kg/h) and 28-day mortality risk. A: 48-hour cumulative NUF, unadjusted Cox model; B: 48-hour cumulative NUF, adjusted for age, sex, presence of septic shock, need for mechanical ventilation, SOFA score, APACHE 11 score, weight-adjusted cumulative fluid balance, creatinine, and lactate; C:72-hour cumulative NUF, unadjusted Cox model; D:72-hour cumulative NUF, adjusted for the same covariates as B. Solid red lines represent hazard ratios, shaded areas indicate 95% confidence intervals.

When analyzed as a continuous variable, the NUF rate showed a nonlinear relationship with 28-day mortality risk in restricted cubic spline analyses (unadjusted *p* for nonlinearity = 0.039; adjusted p for nonlinearity = 0.003; Figure 3(A,B)).

### Secondary outcome

The high-intensity NUF group exhibited significantly higher 60-day mortality (72.6%) compared to moderate- (49.3%) and low-intensity (52.1%) groups (*p* = 0.008, Table 2), with Cox regression indicating higher risk (aHR 1.87, 95% CI 1.21–2.90, high vs moderate, *p* = 0.005; aHR 1.94, 95% CI 1.24–3.04, high vs low, *p* = 0.004, Supplementary Table 2). Despite differences in survival, ΔSOFA scores from baseline to 72 h did not differ significantly between groups (low: +1.15, moderate: +1.25, high: +2.07; *p* = 0.588). High-intensity NUF was associated with markedly negative 72-h fluid balances (−1,368 mL vs. +241 mL/+2,067 mL, *p* <0.001). Among 28-day survivors, trends suggesting lower RRT dependence (47.5% vs. 52.2%) and longer ICU stays (median 31.5 vs. 25 days) in the low-intensity group were not statistically significant (*p* >0.05).

### Sensitivity analysis

Logistic regression analyses adjusting for confounders revealed that high-intensity NUF was independently associated with mortality compared to moderate (adjusted OR [aOR] 2.61, 95% CI 1.24–5.50, *p* = 0.012) and low intensities (aOR 2.73, 95% CI 1.25–5.97, *p* = 0.012), with *p* values remaining significant after Bonferroni correction for multiple comparisons (adjusted threshold: *p* < 0.0167; Supplementary Table 3). A sensitivity analysis incorporating covariates from univariate Cox regression (e.g., Total bilirubin, P/F ratio) into multivariable models produced consistent mortality associations (Supplementary Table 4). Restricted cubic spline (RCS) analysis of 72-h NUF metrics (Figure 3(C–D)) identified a nonlinear relationship between ultrafiltration intensity and mortality risk (*p-nonlinear* = 0.022), with hazard ratios rising steeply above 1.8 mL/kg/h. Further validation using 72-h NUF thresholds demonstrated higher 28-day mortality risks for high-intensity regimens compared to low (aHR 2.01, 95% CI 1.25–3.22, *p* = 0.004) and moderate intensities (aHR 1.74, 95% CI 1.06–2.83, *p* = 0.027). While the 72-h results remained significance after Bonferroni correction (*p* < 0.0167) for the primary comparison (high vs. low intensity), the moderate-to-high contrast approached but did not surpass the adjusted threshold (Supplementary Table 5).

### Subgroup analysis

Among patients with SOFA >8 prior to CRRT, high-intensity NUF was associated with significantly higher 28-day mortality compared to low-intensity (aHR 1.93, 95% CI 1.16–3.23, *p* = 0.012) and moderate-intensity groups (aHR 1.89, 95% CI 1.14–3.12, *p* = 0.013; Table 4). A similar association was observed in the lactate >2 mmol/L subgroup (high vs. low: aHR 1.90, 95% CI 1.05–3.46, *p* = 0.035; high vs. moderate: aHR 2.09, 1.10–3.97, *p* = 0.025), though statistical significance was no longer observed after Bonferroni correction (adjusted threshold: *p* < 0.0167). In septic shock patients, only the comparison between high- and low-intensity NUF remained nominally significant (aHR 1.88, 95% CI 1.12–3.17, *p* = 0.018), which also did not meet the corrected significance threshold. Conversely, no significant associations between NUF intensity and mortality were observed in fluid-overloaded patients (baseline FO ≥5%; high vs. moderate: aHR 1.26, 95% CI 0.68–2.34, *p* = 0.466; high vs. low: aHR 1.04, 95% CI 0.57–1.90, *p* = 0.904).

**Table 4.** Subgroup analysis of 28-day mortality risk by net ultrafiltration intensity: Adjusted Hazard Ratios in critical subpopulations.

Characteristic	Net ultrafiltration intensity	aHR (95% CI)	*p* value
Septic shock^a^	Low vs. Moderate	1.09 (0.62–1.90)	0.775
	High vs. Moderate	1.56 (0.92–2.66)	0.098
	High vs. Low	1.88 (1.12–3.17)	0.018
FO ≥5% prior to CRRT initiation^b^	Low vs. Moderate	1.21 (0.61–2.43)	0.586
	High vs. Moderate	1.26 (0.68–2.34)	0.466
	High vs. Low	1.04 (0.57–1.90)	0.904
SOFA >8 prior to CRRT initiation^c^	Low vs. Moderate	0.98 (0.57–0.68)	0.976
	High vs. Moderate	1.89 (1.14–3.12)	0.013
	High vs. Low	1.93 (1.16–3.23)	0.012
Lactate >2 prior to CRRT initiation^d^	Low vs. Moderate	1.10 (0.56–2.15)	0.788
	High vs. Moderate	2.09 (1.10–3.97)	0.025
	High vs. Low	1.90 (1.05–3.46)	0.035

aHR: adjusted Hazard Ratio; CI: Confidence Interval.

^a^Patients with septic shock adjusted for age, sex, need of mechanical ventilation, SOFA score, APACHE II score, weight-adjusted cumulative fluid balance and creatinine.

^b^Patients with FO ≥ 5% prior to CRRT initiation adjusted for age, sex, presence of septic shock, need of mechanical ventilation, SOFA score and lactate.

^c^Patients with a SOFA score greater than 8 prior to CRRT initiation adjusted for age, sex, presence of septic shock, need of mechanical ventilation, APACHE II score, weight-adjusted cumulative fluid balance, creatinine and lactate.

^d^Patients with lactate level exceeding 2 mmol/L prior to CRRT initiation adjusted for age, sex, presence of septic shock, need of mechanical ventilation, SOFA score and weight-adjusted cumulative fluid balance.

FO, Fluid overload; SOFA, Sequential Organ Failure Assessment.

## Discussion

### Key findings

In critically ill patients with septic AKI requiring CRRT, high-intensity NUF (>1.79 mL/kg/h) was independently associated with markedly higher 28-day mortality compared to low/moderate intensities in the early treatment phase. Restricted cubic spline analysis revealed a J-shaped mortality pattern, showing escalating risks above 1.79 mL/kg/h and an optimal survival window of 1.22–1.79 mL/kg/h. Subgroup analyses further identified significantly higher mortality risks associated with high-intensity NUF among patients with baseline SOFA scores >8 and lactate >2 mmol/L, while no such risks were observed in fluid-overloaded patients. The mortality pattern persisted at 60-day follow-up, while ΔSOFA scores, RRT dependence, and ICU stay duration showed no intergroup differences.

### Comparisons with previous studies

The observed association between higher-intensity NUF (>1.79 mL/kg/h) and increased mortality in this study aligns with previous findings in ICU populations [12,14]. Murugan et al. demonstrated a J-shaped relationship between NUF and mortality across broader critically ill cohorts [9], with a risk inflection point around 1.75 mL/kg/h. Although this study focused on a sepsis-associated AKI subgroup and observed a marginally higher threshold (1.79 mL/kg/h), this variation may be influenced by CRRT-specific management practices (e.g., prescribed NUF targets, modality settings) and ethnic anthropometric differences. Our East Asian cohort exhibited significantly lower body weight (mean 68 kg vs. 80 kg in the RENAL study [12]), potentially leading to weight-adjusted NUF rates being disproportionately influenced by lower lean mass despite comparable absolute fluid removal rates.

Notably, these findings differ from prior research on ultrafiltration intensity (UFNET) and mortality. In studies involving critically ill patients with fluid overload ≥5% requiring RRT [11], UFNET >25 mL/kg/day correlated with survival benefits, whereas this sepsis-AKI cohort (with pre-CRRT body weight-adjusted cumulative fluid balance of 4.46%) showed increased mortality with higher UFNET rates (>1.79 mL/kg/h). These discrepancies require contextual interpretation: reported survival advantages from higher NUF might predominantly apply to populations with marked fluid overload and longer follow-up periods, while our analysis specifically addressed mortality in a demographically distinct sepsis population. These observations suggest that NUF target setting should comprehensively consider patient population characteristics and clinical contexts.

The observed early-phase association pattern between NUF and mortality in septic AKI patients may be linked to pathophysiological features characteristic of this population. Potential mechanisms may include sepsis-associated microcirculatory dysfunction (e.g. glycocalyx degradation and impaired vascular reactivity [21–23], which might lower tolerance to rapid fluid removal. However, the lack of a non-septic control group in this study prevents definitive confirmation of association specificity. Existing literature demonstrates that pathomechanisms underlying acute RRT-associated hypotension—such as impaired vascular tone regulation—have been observed across critically ill populations [24,25], not exclusively in sepsis. This underscores that caution is warranted in attributing observed effects solely to sepsis-specific pathophysiological mechanisms. Our sensitivity analyses demonstrated threshold robustness across 72-h NUF windows, further emphasizing temporal variations in fluid tolerance. The early CRRT phase coincides with peak hemodynamic instability, a period characterized by vascular refilling dynamics that may benefit from incremental NUF adjustments while minimizing inflammation-related hypoperfusion risks [21]. Additionally, the observed mortality nadir at moderate intensity levels corresponds with evolving ‘de-escalation’ strategies, which focus on achieving transitional fluid equilibrium rather than pursuing aggressive fluid removal targets [26].

Our findings suggest the potential value of tailoring NUF rates based on sepsis severity phenotypes, though these observations require validation in prospective studies. In hemodynamically stable patients (SOFA ≤8, lactate ≤2 mmol/L, without shock), moderate-intensity NUF (1.22–1.79 mL/kg/h) emerged as a range of interest for balancing fluid removal and hemodynamic safety in our cohort, providing a basis for future hypothesis testing. For patients with septic shock or high disease severity (SOFA >8, lactate >2 mmol/L), subgroup analyses hint at potential benefits of conservative NUF initiation (<1.22 mL/kg/h) with cautious escalation under monitoring, though this approach remains speculative. The observed J-shaped mortality association reinforces existing evidence from broader CRRT populations regarding the risks of aggressive fluid removal (>1.79 mL/kg/h) during initial therapy. To advance precision fluid management, future trials should prioritize biomarker-guided strategies (e.g., endothelial activation markers [27] and perfusion parameters) to objectively define individualized NUF thresholds, particularly in sepsis subgroups with suspected vascular dysregulation.

### Implications of study findings

This small single-center retrospective study suggests potential risks of aggressive fluid removal strategies during early CRRT in septic AKI, demonstrating a nonlinear association between ultrafiltration intensity and mortality outcomes. Elevated early-phase ultrafiltration rates may correlate with adverse outcomes, particularly in sepsis patients with microcirculatory dysfunction. While these findings raise questions about the universal application of high-intensity net ultrafiltration, they should be interpreted cautiously given the study’s observational design and limited sample size.

This nonlinear risk pattern aligns with broader evidence advocating hemodynamic stability over maximal fluid clearance in critical care populations. However, the hypothesis-generating nature of these data precludes definitive clinical recommendations. Clinicians might consider individualized risk assessments incorporating dynamic markers of shock and inflammation when modulating ultrafiltration rates. Our results suggest the need for adaptive strategies, but such strategies would require validation through multicenter trials comparing protocolized versus physiology-guided approaches. Until conclusive evidence emerges, our results suggest that continuous reassessment of fluid balance during CRRT may be superior to use of fixed NUF targets.

### Study strengths and limitations

This investigation represents the first exploration of associations between early net ultrafiltration dynamics and clinical outcomes in patients with septic acute kidney injury receiving continuous renal replacement therapy. Its methodological rigor is reinforced through multivariable adjustments and several sensitivity analyses.

We acknowledge the following limitations to this study. The retrospective design precluded the systematic capture of longitudinal hemodynamic parameters, particularly echocardiographic assessments of cardiac function, which could influence ultrafiltration tolerance. We addressed this limitation by incorporating surrogate markers of hemodynamic stress, including MAP and biochemical profiles. Additionally, temporal granularity regarding sepsis diagnosis-to-CRRT intervals was unavailable, potentially confounding the relationship between disease phase and ultrafiltration aggressiveness. Future prospective studies should standardize sepsis timeline documentation alongside advanced hemodynamic monitoring. The single-center nature restricts generalizability, though baseline patient characteristics align with broader critical care populations. While residual confounding due to unmeasured variables (e.g., intradialytic blood pressure fluctuations) remains possible, key illness severity indices were rigorously adjusted for in the analyses. Notably, the observed associations should be interpreted as hypothesis-generating rather than indicative of causality, necessitating validation through interventional trials.

These constraints notwithstanding, the study advances current understanding by characterizing sepsis-specific risks of ultrafiltration strategies, providing a framework for individualized fluid management in this vulnerable population.

## Conclusion

A high-intensity net ultrafiltration (NUF >1.79 mL/kg/h) during early CRRT was independently associated with a higher 28-day mortality risk compared to lower rates in patients with septic AKI. Analysis revealed a nonlinear relationship between NUF intensity and mortality, with moderate-intensity regimens (1.22–1.79 mL/kg/h) demonstrating the lowest mortality rate (43.8%). Mortality risk was associated with progressively higher NUF intensities, particularly in patients with SOFA scores >8 and lactate levels >2 mmol/L. These findings suggest individualized fluid management strategies prioritizing hemodynamic stability may be preferred over aggressive fluid removal in critically ill septic AKI populations. However, this approach requires validation in prospective studies to confirm its clinical benefits and refine optimal NUF thresholds.

## Ethics approval and consent to participate

This study was approved by Bioethics Committee of Beijing Friendship Hospital and informed consent was waived. (2020-P2-267-02).

## Consent for publication

Not applicable.

## Supplementary Material

Supplementary_file - Clean.docx

## Data Availability

The datasets used and/or analyzed during the current study are available from the corresponding author on reasonable request.
