# Supplementary material for: Early net ultrafiltration thresholds and mortality in critically ill patients with septic acute kidney injury receiving continuous renal replacement therapy
Source: Ren Fail. 2025 May 29;47(1):2511277. doi: 10.1080/0886022X.2025.2511277 (PMC12123948; doi:10.1080/0886022X.2025.2511277)
Supplement: Supplementary_file - Clean.docx [file IRNF_A_2511277_SM5389.docx]

**Supplementary File**

[Supplementary Table 1: Comparison of Baseline Characteristics by Survival Status 2](#_Toc191224404)

[Supplementary Table 2: Univariate and Multivariable Cox Proportional Hazards Regression Analysis of 60-Day All-Cause Mortality 4](#_Toc191224405)

[Supplementary Table 3: Association of Net Ultrafiltration Intensity with 28-Day Mortality: Univariable and Multivariable Logistic Regression Analyses 5](#_Toc191224406)

[Supplementary Table 4: Association of Net Ultrafiltration Intensity with 28-Day Mortality: A Cox Model Adjusted for Significant Predictors 6](#_Toc191224407)

[Supplementary Table 5: Association of Net Ultrafiltration Intensity with 28-Day Mortality at 72 Hours After CRRT Initiation: Univariate and Multivariable Cox Regression Analyses 7](#_Toc191224408)

# Supplementary Table 1: Comparison of Baseline Characteristics by Survival Status

| **Variables** | **Total**  **(n = 219)** | **Survived**  **(n = 104)** | **Non-survived**  **(n = 115)** | ***p* value** |
| --- | --- | --- | --- | --- |
| Age (years) | 66.3 ± 18.6 | 64.4 ± 18.7 | 68.0 ± 18.5 | 0.096 |
| Male n (%) | 144 (65.8) | 75 (72.1) | 69 (60.0) | 0.065 |
| Weight (kg) | 68.2 ± 16.7 | 69.2 ± 14.2 | 67.3 ± 18.7 | 0.126 |
| BMI (kg/m^2^) | 24.0 ± 4.7 | 24.2 ± 4.3 | 23.8 ± 5.1 | 0.285 |
| Baseline Cr (μmol/L) | 81.6 (64.0, 109.4) | 84.1 (69.3, 124.6) | 76.3 (59.9, 103.2) | 0.028 |
| Comorbidity, n (%) |  |  |  |  |
| Hypertension | 86 (39.3) | 35 (33.7) | 51 (44.3) | 0.128 |
| Cardiac disease | 67 (30.6) | 28 (26.9) | 39 (33.9) | 0.305 |
| Diabetes | 77 (35.2) | 41 (39.4) | 36 (31.3) | 0.257 |
| Chronic kidney disease | 55 (25.1) | 29 (27.9) | 26 (22.6) | 0.436 |
| Admission source, n (%) |  |  |  | 0.725 |
| Emergency | 72 (32.9) | 35 (33.7) | 37 (32.2) |  |
| Medical ward | 80 (36.5) | 40 (38.5) | 40 (34.8) |  |
| Surgical ward | 60 (27.4) | 25 (24.0) | 35 (30.4) |  |
| Transfer from another hospital | 7 (3.2) | 4 (3.8) | 3 (2.6) |  |
| Site of infection, n (%) |  |  |  | 0.284 |
| Respiratory | 156 (71.2) | 67 (64.4) | 89 (77.4) |  |
| Digestive | 36 (16.4) | 21 (20.2) | 15 (13.0) |  |
| Urinary | 19 (8.7) | 11 (10.6) | 8 (7.0) |  |
| Blood | 4 (1.8) | 2 (1.9) | 2 (1.7) |  |
| Other | 4 (1.8) | 3 (2.9) | 1 (0.9) |  |
| Indications for CRRT, n(%) |  |  |  | 0.022 |
| Fluid overload | 50 (22.8) | 20 (19.2) | 30 (26.1) |  |
| acid-base disorder | 11 (5.0) | 8 (7.7) | 3 (2.6) |  |
| electrolyte disorder | 15 (6.8) | 9 (8.7) | 6 (5.2) |  |
| Severe non-renal organ dysfunction | 20 (9.1) | 9 (8.7) | 11 (9.6) |  |
| Oliguria | 84 (38.4) | 47 (45.2) | 37 (32.2) |  |
| Other | 39 (17.8) | 11 (10.6) | 28 (24.3) |  |
| AKI stage at CRRT initiation, n (%) |  |  |  | 0.122 |
| 1 | 46 (21.0) | 20 (19.2) | 26 (22.6) |  |
| 2 | 49 (22.4) | 18 (17.3) | 31 (27.0) |  |
| 3 | 124 (56.6) | 66 (63.5) | 58 (50.4) |  |
| SOFA score | 10.7 ± 3.9 | 9.9 ± 3.9 | 11.5 ± 3.9 | 0.003 |
| APACHE II score | 24.4 ± 6.8 | 23.1 ± 6.1 | 25.7 ± 7.3 | 0.024 |
| Mechanical ventilation,  n (%) | 153 (69.9) | 59 (56.7) | 94 (81.7) | <0.001 |
| Septic shock, n (%) | 141 (64.4) | 58 (55.8) | 83 (72.2) | 0.016 |
| Time from ICU admission to CRRT initiation (d) | 1 (0, 4) | 1 (0, 1.8) | 1 (0, 8.0) | 0.108 |
| 24-hour urine before CRRT initiation (mL) | 442 (250, 720) | 442 (276, 761） | 445 (250, 695) | 0.672 |
| CFB prior to CRRT (mL) | 3098  (2040, 4734) | 2463  (1812, 3819) | 3815  (2233, 5260) | 0.001 |
| Weight-adjusted CFB (%) prior to CRRT | 4.46 (2.99, 7.87) | 3.71 (2.78, 6.80) | 5.64 (3.51, 8.46) | 0.001 |
| MAP at CRRT initiation | 82.6 ± 13.1 | 84.2 ± 12.7 | 81.2 ± 13.3 | 0.063 |
| Laboratory before CRRT |  |  |  |  |
| WBC (×10^9^/L) | 11.7 (6.7, 16.7) | 11.9 (7.1, 19.3) | 11.7 (6.1, 15.7) | 0.284 |
| Hemoglobin (g/L) | 84.0 (72.0, 105.0) | 88.0 (70.5, 112.8) | 84.0 (72.0, 97.0) | 0.182 |
| Platelets (×10^9^/L) | 98.0 (56.0, 164.0) | 107.0 (63.0, 180.8) | 89.0 (52.0, 159.0) | 0.193 |
| Serum sodium (mmol/L) | 142.8 ± 8.4 | 141.6 ± 8.1 | 143.9 ± 8.6 | 0.060 |
| Serum potassium (mmol/L) | 4.66 ± 0.71 | 4.60 ± 0.70 | 4.72 ± 0.73 | 0.234 |
| Serum potassium ≥5.5mmol/L | 31 (14.2) | 12 (11.5) | 19 (16.5) | 0.335 |
| Creatinine (μmol/L) | 263.5  (165.3, 431.2) | 306.9  (216.6, 493.9) | 222.8  (151.4, 352.7) | <0.001 |
| Creatinine ≥300μmol/L | 90 (41.1) | 55 (52.9) | 35 (30.4) | 0.001 |
| Total bilirubin (μmol/L) | 17.8 (10.2, 41.0) | 17.6 (9.3, 34.5) | 17.9 (11.5, 76.6) | 0.043 |
| Albumin (g/L) | 26.6 ± 8.7 | 27.7 ± 11.6 | 25.8 ± 4.7 | 0.120 |
| Procalcitonin (ng/mL) | 4.6 (1.3, 12.4) | 6.2 (1.3,19.7) | 4.1 (1.3, 9.9) | 0.152 |
| pH value | 7.36 ± 0.08 | 7.37 ± 0.08 | 7.35 ± 0.09 | 0.157 |
| pH ≤ 7.2 | 11 (5.0) | 6 (5.8) | 5 (4.3) | 0.760 |
| P/F ratio (mmHg) | 188 (127, 275) | 213 (113, 300) | 161 (106, 257) | 0.008 |
| Lactate (mmol/L) | 1.9 (1.2, 3.4) | 1.6 (1.0, 3.0) | 2.3 (1.4, 4.1) | 0.022 |
| NT-proBNP (pg/mL) | 8229  (1859, 28399) | 8580  (1898, 25454) | 10123  (2450, 28387) | 0.841 |
| NUF rate (mL/kg/h) | 1.48 (1.05, 2.04) | 1.36 (1.03,1 .70) | 1.61 (1.12, 2.38) | 0.011 |

BMI, Body Mass Index; SOFA, Sequential Organ Failure Assessment; APACHE, Acute Physiology and Chronic Health Evaluation; ICU, Intensive Care Unit; CRRT, Continuous Renal Replacement Therapy; CFB, Cumulative Fluid Balance; MAP, Mean Arterial Pressure; WBC, White Blood Cells; P/F ratio, PaO₂/FiO₂ ratio; NUF, Net Ultrafiltration; Continuous variables are expressed as mean ± SD or median [Q1, Q3] and nominal variables as n (%)

# Supplementary Table 2: Univariate and Multivariable Cox Proportional Hazards Regression Analysis of 60-Day All-Cause Mortality

| Net ultrafiltration intensity | HR (95% CI) | *p* value | aHR(95%CI) | *p* value |
| --- | --- | --- | --- | --- |
| Low vs Moderate | 1.07 (0.68–1.68) | 0.786 | 0.97 (0.60–1.55) | 0.885 |
| High vs Moderate | 1.98 (1.30–3.03) | 0.002 | 1.87 (1.21–2.90) | 0.005 |
| High vs Low | 1.90 (1.22–2.82) | 0.004 | 1.94 (1.24–3.04) | 0.004 |

HR: Hazard Ratio; aHR: adjusted Hazard Ratio; CI: Confidence Interval

Adjusted for age, gender, presence of septic shock, need of mechanical ventilation, SOFA score, APACHE II score, weight-adjusted CFB, creatinine and lactate.

# Supplementary Table 3: Association of Net Ultrafiltration Intensity with 28-Day Mortality: Univariable and Multivariable Logistic Regression Analyses

| Net ultrafiltration intensity | OR (95% CI) | *p* value | aOR (95%CI) | *p* value |
| --- | --- | --- | --- | --- |
| Low vs Moderate | 1.06 (0.55–2.03) | 0.868 | 0.96 (0.46–1.98) | 0.904 |
| High vs Moderate | 2.79 (1.42–5.48) | 0.003 | 2.61 (1.24–5.50) | 0.012 |
| High vs Low | 2.64 (1.34–5.18) | 0.005 | 2.73 (1.25–5.97) | 0.012 |

OR: Odds Ratio; aOR: adjusted Odds Ratio; CI: Confidence Interval

Adjusted for age, gender, presence of septic shock, need of mechanical ventilation, SOFA score, APACHE II score, weight-adjusted CFB, creatinine and lactate.

# Supplementary Table 4: Association of Net Ultrafiltration Intensity with 28-Day Mortality: A Cox Model Adjusted for Significant Predictors

| Variables | Unit | Univariate model | | Multivariable model | |
| --- | --- | --- | --- | --- | --- |
|  |  | HR (95%CI) | *p* value | HR (95%CI) | *p* value |
| Net ultrafiltration |  | **–** | **–** | **–** | **–** |
| Low-intensity | vs. Moderate-intensity | 1.03 (0.63–1.67) | 0.922 | 1.09 (0.65–1.84) | 0.738 |
| High-intensity | vs. Moderate-intensity | 2.02 (1.30–3.15) | 0.002 | 1.93 (1.22–3.06) | 0.005 |
| Gender | Male vs. Female | 1.48 (1.02–2.15) | 0.039 | 1.22 (0.80–1.84) | 0.358 |
| Septic Shock | Yes vs. No | 1.72 (1.13–2.59) | 0.009 | 0.98 (0.60–1.59) | 0.977 |
| Mechanical ventilation | Yes vs. No | 2.42 (1.51–3.89) | < 0.001 | 1.69 (0.92–2.80) | 0.093 |
| SOFA score | per 1 point increase | 1.07 (1.02–1.12) | 0.003 | 1.03 (0.96–1.07) | 0.645 |
| APACHE II score | per 1 point increase | 1.04 (1.01–1.07) | 0.005 | 1.03 (0.99–1.06) | 0.115 |
| Weight-adjusted CFB (%) | per 1%. increase | 1.06 (1.02–1.11) | 0.005 | 1.01 (0.96–1.07) | 0.630 |
| Serum sodium | per 10mmol/L increase | 1.03 (1.00–1.05) | 0.026 | 1.16 (0.91–1.49) | 0.224 |
| Creatinine | per 10μmol/L increase | 0.98 (0.97–0.99) | < 0.001 | 0.99 (0.98–1.00) | 0.050 |
| Total bilirubin | per 10μmol/L increase | 1.03 (1.02–1.05) | < 0.001 | 1.02 (1.01–1.04) | 0.003 |
| PaO₂/FiO₂ ratio | per 10mmHg increase | 0.98 (0.96–0.99) | 0.009 | 0.99 (0.97–1.01) | 0.201 |
| Lactate | per 1mmol/L increase | 1.06 (1.01–1.11) | 0.017 | 1.02 (0.96–1.07) | 0.580 |

SOFA, Sequential Organ Failure Assessment; APACHE, Acute Physiology and Chronic Health Evaluation; HR: Hazard Ratio; CI: Confidence Interval

Variables included in the multivariable Cox model were selected based on univariate regression analysis with a significance threshold of p < 0.1. The final model retained predictors with the smallest p-values from the univariate screening.

# Supplementary Table 5: Association of Net Ultrafiltration Intensity with 28-Day Mortality at 72 Hours After CRRT Initiation: Univariate and Multivariable Cox Regression Analyses

| Net ultrafiltration intensity | HR (95% CI) | *p* value | aHR (95%CI) | *p* value |
| --- | --- | --- | --- | --- |
| Low vs Moderate | 1.30 (0.79–2.11) | 0.301 | 1.04 (0.63–1.73) | 0.879 |
| High vs Moderate | 2.19 (1.39–3.47) | 0.001 | 1.74 (1.06–2.83) | 0.027 |
| High vs Low | 1.97 (1.27–3.06) | 0.003 | 2.01 (1.25–3.22) | 0.004 |

HR: Hazard Ratio; aHR: adjusted Hazard Ratio; CI: Confidence Interval

Adjusted for age, gender, presence of septic shock, need of mechanical ventilation, SOFA score, APACHE II score, weight-adjusted CFB , creatinine and lactate.
